# Untargeted Metabolomics Reveals the Potential Antidepressant Activity of a Novel Adenosine Receptor Antagonist

**DOI:** 10.3390/molecules27072094

**Published:** 2022-03-24

**Authors:** Arnold Petrus Smith, Jeremie Zander Lindeque, Mietha Magdalena van der Walt

**Affiliations:** Human Metabolomics, Faculty of Natural and Agricultural Sciences, Potchefstroom Campus, North-West University, Private Bag x6001, Box 269, Potchefstroom 2531, South Africa; arno.smith19@gmail.com (A.P.S.); zander.lindeque@nwu.ac.za (J.Z.L.)

**Keywords:** adenosine receptor, antagonist, depression, untargeted metabolomics, ^1^H-NMR, GC-TOFMS, KW-6002, imipramine

## Abstract

Depression is the most common mental illness, affecting approximately 4.4% of the global population. Despite many available treatments, some patients exhibit treatment-resistant depression. Thus, the need to develop new and alternative treatments cannot be overstated. Adenosine receptor antagonists have emerged as a promising new class of antidepressants. The current study investigates a novel dual A_1_/A_2A_ adenosine receptor antagonist, namely 2-(3,4-dihydroxybenzylidene)-4-methoxy-2,3-dihydro-1H-inden-1-one (**1a**), for antidepressant capabilities by determining its metabolic profiles and comparing them to those of two reference compounds (imipramine and KW-6002). The metabolic profiles were obtained by treating male Sprague-Dawley rats with **1a** and the reference compounds and subjecting them to the forced swim test. Serum and brain material was consequently collected from the animals following euthanasia, after which the metabolites were extracted and analyzed through untargeted metabolomics using both ^1^H-NMR and GC-TOFMS. The current study provides insight into compound **1a**’s metabolic profile. The metabolic profile of **1a** was similar to those of the reference compounds. They potentially exhibit their antidepressive capabilities via downstream effects on amino acid and lipid metabolism.

## 1. Introduction

Depression, sometimes described as the illness of our times, is one of the world’s most prevalent diseases, affecting an estimated 322 million people worldwide, nearly 4.4% of the global population [1]. According to the 11th Revision of the International Classification of Diseases and Related Health Problems (ICD), depression is documented as the most common mental disorder in the global population. It is recognized as one of the main contributors to the worldwide burden of disease [2]. Much of this burden stems from depression’s ability to make patients lose interest in what makes them happy and give them a lasting feeling of sadness, among a wide range of other possible physical and mental symptoms [3]. The ongoing pandemic that we find ourselves in has only served to exacerbate this burden, with Santomauro et al. [4] estimating an average increase of 27.6% in global depression cases.

An extensive review of treatment management of depression is described by Gautam et al. [5]. Treatment options are divided into antidepressant drugs, electroconvulsive therapy, and psychosocial interventions. Numerous antidepressant drugs are available, and considering their safety profiles and side effects, selective serotonin reuptake inhibitors (SSRIs) are generally considered first-line antidepressant drugs. Tricyclic antidepressants, mirtazapine, bupropion, and venlafaxine are other preferred choices [5]. However, despite this wide selection of existing treatments, some patients still exhibit treatment-resistant depression [6]—for example, Parkinson’s Disease (PD)-related depression (a co-morbidity) [7,8]. Accordingly, the need to develop and investigate alternative therapies cannot be overstated.

Adenosine receptor antagonists are a new class of antidepressants that has shown promise in recent years. Adenosine and its derivatives have been used clinically since the 1940s [9]. Adenosine regulates physiological actions through extracellular G-protein-coupled receptors called adenosine receptors (ARs), of which four AR subtypes (A_1_, A_2A_, A_2B_, and A_3_) exist. Of these, the A_1_ and A_2A_ ARs have attracted the most attention in recent years as potential drug targets for various diseases, ranging from PD (A_1_ and A_2A_) [10] and Alzheimer’s Disease (A_1_) [11], to diabetes (A_1_ and A_2A_) [12], certain cancers (A_1_ and A_2A_) [13], and depression (A_2A_) [14]. In addition to this, the A_1_ and A_2A_ ARs are the most expressed of the ARs in the brain [15]. Activation of ARs has been shown to induce depression-like states in various animal models. Thus, one possible antidepressive mechanism of action of AR antagonists is via the direct reduction in AR activation. However, the exact mechanism is still unclear, with various hypotheses previously described [16].

The antidepressant capabilities of AR antagonists have been shown to exist in quite a few cases. Caffeine is a well-known example of a non-selective A_1_ and A_2A_ AR antagonist (A_1_*K_i_* = 55 μM; A_2A_*K_i_* = 50 μM) [17], and epidemiological studies have shown a strong relationship between high coffee (i.e., caffeine) consumption and a reduced risk of developing depression [18]. Furthermore, low doses of caffeine may potentiate the effects of classical antidepressants such as desipramine, imipramine, duloxetine, fluoxetine, and paroxetine [18].

Inspired by the basic xanthine scaffold of caffeine, numerous AR antagonists have been developed by various research groups, with high affinity towards the A_1_ and A_2A_ AR sub-types. The first clinically approved A_2A_ AR antagonist, KW-6002, was initially developed to treat PD. However, it was later shown to improve PD-related depression while also exhibiting antidepressant-like effects in two animal models of depression: the forced swim test (FST) and the tail suspension test [15]. Furthermore, when DPCPX (dipropylcyclopentylxanthine, a selective A_1_ AR antagonist) was co-administered with certain SSRIs/norepinephrine reuptake inhibitors (namely imipramine, escitalopram, and reboxetine), it improved their antidepressant effects [19]. Building on the latter, it is worth investigating AR antagonists as an alternative therapy for depression.

Given the antidepressant activity attributed to A_1_ and A_2A_ AR antagonism, a dual A_1_/A_2A_ AR antagonist may possess antidepressant capabilities, warranting further exploration. In part, the flavonoid derivative 5,3′-dihydroxyflavone, previously documented with A_1_ and A_2A_ AR affinity (A_1_*K_i_* (rat) = 0.956 μM and A_2A_*K_i_* (rat) = 1.44 μM), served as inspiration for investigating the benzylidene tetralones as a novel AR drug class [20]. Modifications to the benzylidene tetralone scaffold resulted in (*E*)-5-hydroxy-2-(3-hydroxybenzylidene)-3,4-dihydronaphthalen-1(*2H*)-one possessing AR affinity in the low micromolar range (A_1_*K_i_* (rat) = 1.62 μM and A_2A_*K_i_* (rat) = 5.46 μM) [21]. In analogy to (*E*)-5-hydroxy-2-(3-hydroxybenzylidene)-3,4-dihydronaphthalen-1(*2H*)-one, further structural optimization via hydroxy-substituted 2-benzylidene-1-indanone analogs [22] and methoxy-substituted 2-benzylidene-1-indanone analogs [23] was investigated as promising novel A_1_ and A_2A_ AR antagonists. It seemed that C4 hydroxy substitution on ring A of the benzylidene indanones, combined with meta (C3′) and para (C4′) dihydroxy substitution on ring B, resulted in *K_i_* values for both the A_1_ and A_2A_ AR below 1 μM. Of note, (*E*)-2-(3,4-dihydroxybenzylidene)-4-hydroxy-2,3-dihydro2,3-dihydro-1H-inden-1-one (A_1_ *K_i_* (rat) = 0.435 μM; A_2A_ *K_i_* (rat) = 0.903 μM) was further optimized to obtain the methoxy-substituted 2-benzylidene-1-indanone (*E*)-2-(3,4-dihydroxybenzylidene)-4-methoxy-2,3-dihydro-1H-inden-1-one (compound **1a**), which possessed dual A_1_ and A_2A_ AR affinity in the nanomolar range (A_1_ *K_i_* (rat) = 0.042 μM; A_2A_ *K_i_* (rat) = 0.078 μM) [23]. Thus, C4 methoxy substitution on ring A, together with meta (3′) and para (4′) dihydroxy substitution on ring B, resulted in improved AR affinity [23].

Based on the above, one potential candidate worth investigating further is the novel methoxy-substituted benzylidene indanone derivative and dual A_1_/A_2A_ AR antagonist, compound **1a**. Unlike the xanthine derivatives caffeine, KW-6002, and DPCPX, compound **1a** represents a novel non-xanthine class of AR antagonists [23].

To our knowledge, the elucidation and comparison of the metabolic profiles of serum, whole brain (excluding striata), and striata after treatment of **1a** in a forced swim test (FST) animal model has not been studied previously. The metabolic profiles of **1a** are potential indicators of interest for systemic (via serum) and neuronal (via brain) antidepressant metabolic profiles after treatment with a dual A_1_/A_2A_ AR antagonist in an FST.

**Figure 1:**
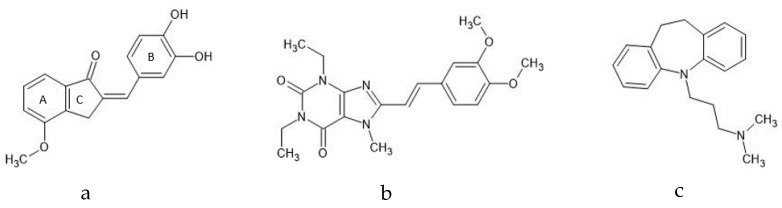
Chemical structures of the test compound (**a**) 2-(3,4-dihydroxybenzylidene)-4-methoxy-2,3-dihydro-1H-inden-1-one (compound **1a**) and reference compounds (**b**) KW-6002 and (**c**) imipramine.

In the current study, we hypothesized that the metabolic profiles of **1a** based on serum, whole brain (excluding striata), and striata might exhibit similar metabolite changes to one or both the reference compounds after treatment. This study aimed to reveal the metabolic profiles of **1a** and reference compounds (KW-6002 and imipramine) based on serum, whole-brain (excluding striata), and striata samples by combining the results of GC-TOFMS and ^1^H-NMR and investigating the relationship between their metabolic profiles to gain insight into **1a**’s potential molecular mechanisms (Figure 1).

## 2. Results

### 2.1. Compound ***1a***’s Metabolic Profile

Following processing and statistical analysis of the data acquired from both the ^1^H-NMR and GC-TOFMS analyses, the test compound (**1a**) was found to have induced statistically significant alterations in 49 metabolites’ concentrations across the three examined matrices. The identified metabolites, their associated metabolism, and the matrix and direction of alteration are presented in Table 1. Of the 49 identified metabolites, 16 are associated with the amino acid metabolic pathways, 19 with lipid metabolism, and four with central carbon metabolism. Significant differences were found in 20 metabolites present in the serum samples. In turn, noteworthy differences in 19 metabolites were identified in the brain samples (excluding the striata) and 22 in the striata “only” samples. Overall, the striata and serum samples exhibited similar metabolic alterations, with most of their observed metabolites being decreased. In contrast, most of the metabolites observed in the brain samples (excluding the striata) were increased.

### 2.2. KW-6002’s Metabolic Profile

As mentioned in Section 1, KW-6002 is a selective A_2A_ AR antagonist. Thirty metabolites presented statistically significant alterations in the group treated with KW-6002, with 14 of these observed in the serum samples, 15 in the whole-brain samples (excluding striata), and two in the striata. Similar to compound **1a**, amino acid and lipid metabolism appear most affected by the treatment, presenting 10 and 13 altered metabolites, respectively. Furthermore, the serum and striata samples again exhibited similar alteration patterns, with the majority of the metabolite levels being decreased, while the whole brains (excluding striata) mostly displayed increased alterations. The complete list of metabolites and additional information can be found in Table 2.

### 2.3. Imipramine Metabolic Profile

Among compound **1a** and the reference compounds, imipramine displayed 34 statistically significant metabolites. Eight were observed in the serum samples, 19 in the whole brain (excluding striata) samples, and 17 in the striata samples. In analogy to compound **1a** and KW-6002, amino acid and lipid metabolism presented the most significant changes in metabolite levels, totaling 6 and 18 metabolites, respectively. In addition, central carbon metabolism showed some alteration with four metabolites. Furthermore, as documented in Table 3, most of the serum and striata samples’ exhibited decreased metabolite levels, while the whole-brain (excluding striata) samples were found to present primarily increased alterations. This is again consistent with the observations made for compound **1a** and KW-6002.

### 2.4. Metabolite Distribution within Serum, Whole Brain (Excluding Striata), and Striata

**Figure 2:**
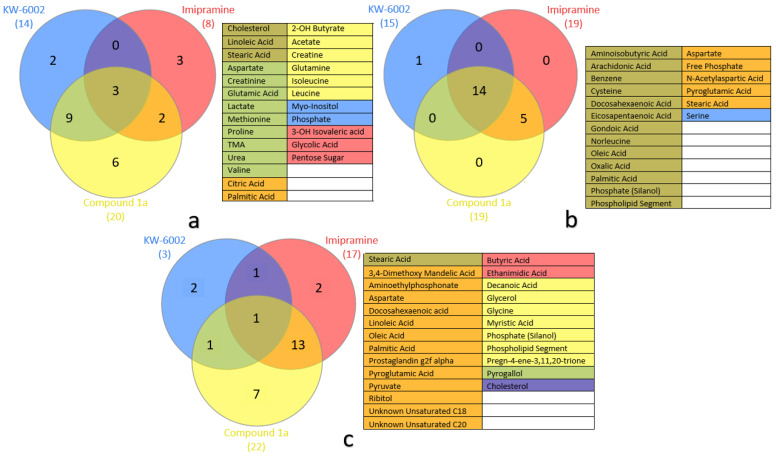
Venn diagrams illustrating the distribution of altered metabolites in (**a**) serum, (**b**) whole brain (excluding striata), and (**c**) striata.

Figure 2 shows Venn diagrams illustrating the altered metabolites together with the treatment that induced the alteration in serum, whole brain (excluding striata), and striata. Evaluating the altered metabolites in serum (see Figure 2a), compound **1a** totaled 20 metabolites, KW-6002 totaled two metabolites, and imipramine totaled eight metabolites. The three treatments (compound **1a**, KW-6002, and imipramine) did not show considerable overlap in affected metabolites in the serum samples. The overlap metabolites included: cholesterol, linoleic acid, and stearic acid. Overall, compound **1a** and KW-6002 uniquely overlapped with nine metabolites. Furthermore, compound **1a** presented six unique metabolites (2-hydroxybutyrate, acetate, creatinine, glutamine, isoleucine, and leucine) compared to KW-6002 and imipramine.

Assessing the altered metabolites in the whole brain (excluding striata), compound **1a** totaled five metabolites, KW-6002 totaled 15 metabolites, and imipramine totaled 19 metabolites. At least two treatments affected most of the altered metabolites in the whole brain (excluding striata). The majority were affected by compound **1a**, KW-6002, and imipramine, which overlapped with 14 metabolites. Of these, five metabolites were unique to compound **1a** and imipramine (see Figure 2b).

Finally, as illustrated in Figure 2c, most of the alterations seen in the striata were induced by compound **1a** and imipramine, with considerable overlap between them (14 metabolites), while KW-6002 had seemingly little effect. Only one metabolite, pyrogallol, overlapped with compound **1a**. Overall, compound **1a** totaled 22 metabolites, KW-6002 totaled two, and imipramine totaled 17 metabolites.

### 2.5. General Observations

**Figure 3:**
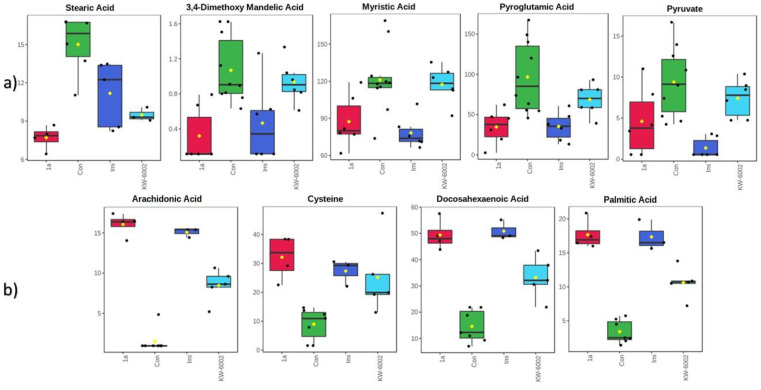
Box-plots illustrating the (**a**) decreased concentrations in serum (stearic acid) and striata (3,4-dimethoxy mandelic acid, myristic acid, pyroglutamic acid, and pyruvate) metabolites and (**b**) the increased concentrations in whole-brain (excluding striata) (arachidonic acid, cysteine, docosahexaenoic acid, and palmitic acid) metabolites.

When considering the results as a whole, it becomes clear that all of the treatments (compound **1a**, KW-6002, and imipramine) exhibited overlapped metabolites among their metabolic profiles. As mentioned throughout Section 2.1, Section 2.2 and Section 2.3, this came down to statistically significant differences between each treatment group and the control group, mostly pertaining to amino acid and lipid metabolism, and central carbon metabolism to an extent. Furthermore, in all three treatments (compound **1a**, KW-6002, and imipramine), serum and striata samples showed similar alterations (mostly decreased), while the whole-brain (excluding striata) samples displayed mainly increases in metabolite levels (see Figure 3).

## 3. Discussion

This study assessed changes in serum, whole-brain (excluding striata), and striata metabolites in an FST animal model where animals were either untreated (control group) or treated with compound **1a** or reference compounds. The selective A_2A_ AR antagonist KW-6002 and tricyclic antidepressant imipramine served as reference compounds.

Our major findings are the significant alterations of amino acid and lipid metabolism in all three treatment groups (compound **1a**, KW-6002, and imipramine) in comparison to the untreated vehicle control group. In addition, some influence on central carbon metabolism was noted. As will be discussed in more detail below, we propose that the mentioned metabolic pathways are related to general energy metabolism. Furthermore, since KW-6002 is known to possess antidepressant properties [18,25] and imipramine is a well-known antidepressant, the similar metabolic profiles that the reference compounds share with **1a** may indicate the test compound’s potential antidepressant effect.

### 3.1. Systemic Metabolic Profiles—Via Serum

The systemic metabolic profiles were constructed via untargeted metabolomics of the serum samples. In our studies, a total of 25 metabolites in serum were detected using both ^1^H-NMR and GC-TOFMS analysis. Even though compound **1a** was evaluated for its potential antidepressant capabilities, information regarding its systemic effects remains necessary since **1a** must pass through the rest of the bodily systems to reach the brain. The latter information is important with AR antagonists, given their vast distribution throughout the body [26]. Despite this, no concerning observations were made during compound **1a**’s systemic metabolic profile analysis.

**Figure 4:**
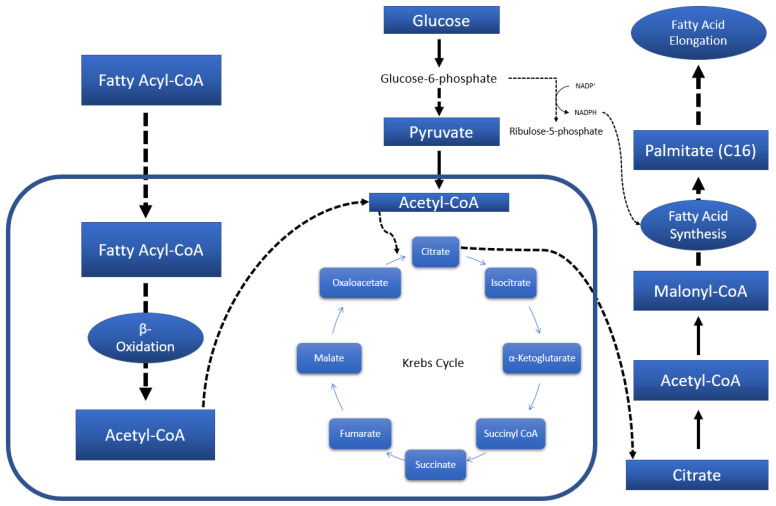
Brief representation of the main fatty acid metabolic pathways.

Statistically significant differences between the metabolites of the control group and treatment groups (compound **1a**, KW-6002, and imipramine) indicate an association with energy metabolism. In accordance, perturbations in energy metabolism have previously been documented in a depression study [27]. Foremost to general energy metabolism is, of course, glycolysis, and central carbon metabolism by extension. Alternatively, when glucose metabolism is insufficient to supply the body’s energy demands, lipids may be catabolized to address the deficit, as illustrated in Figure 4. Finally, as a last resort, certain amino acids can also be metabolized to meet energy demands should the deficit persist.

Amino acid metabolism, lipid metabolism, and, to a lesser extent, central carbon metabolism are the three most prominently affected systemic metabolic pathways among compound **1a**, KW-6002, and imipramine, indicating possible remediation of perturbed energy metabolism.

When considering the results (Section 2), the first indication of perturbed energy metabolism is the decreased lactate levels in rats treated with compound **1a** and KW-6002. Lactate is produced when glucose is metabolized anaerobically [28], i.e., when the Krebs cycle functions insufficiently and oxygen is unavailable. Reduced lactate levels could thus point to improved Krebs functioning. Furthermore, the treatment groups displayed reduced stearic acid (**1a**, KW-6002, and imipramine), palmitic acid (**1a** and imipramine), and cholesterol (**1a**, KW-6002, and imipramine). Stearic and palmitic acid are both central intermediates of the fatty acid biosynthesis pathway, while cholesterol is synthesized from Acetyl-CoA, a metabolic end-product of fatty acid oxidation. Reduction in these metabolites could thus indicate reduced fatty acid biosynthesis and oxidation, which in turn may well signify reduced reliance on fatty acids for energy. Finally, though not as prominent across all treatment groups, compound **1a** exhibited reduced levels of the amino acids that can be catabolized to Krebs cycle intermediates, namely leucine, isoleucine, and valine. In addition, KW-6002 also showed reduced valine (see Figure 2a). Zhao et al. [29] documented increased levels of leucine and valine within the hippocampus after treatment with imipramine in an FST [29]. In the current study, reduced levels of leucine and valine were only exhibited by compound 1a’s metabolic profile in serum. Leucine and valine were not among the statistically significant metabolites for imipramine in either serum, whole brain (excluding striata), or striata. This discrepancy with Zhao et al.’s findings may be ascribed to the different brain region that was used for our study.

Consequently, it is possible that amino acids were being recruited to supplement energy metabolism in the control group (which was also subjected to the FST), but the specified treatments (compound **1a**, KW-6002, and imipramine) were able to restore the energy metabolism to a degree where this was no longer necessary. In addition to this, increased creatine and creatinine levels also indicate energy metabolism dysregulation [30]. Again, compound **1a** showed reduced levels of both of these metabolites, while KW-6002 exhibited reduced creatinine levels, further supporting the idea of restored energy metabolism.

### 3.2. Neuronal Metabolic Alterations—Via Brain Samples

Based on KW-6002 being included as a reference compound, and being a selective A_2A_ AR antagonist, it was decided to dissect the whole-brain tissue into the striata (expressing A_2A_ AR subtypes) and the remaining whole brain (excluding the striata and consequently mainly expressing A_1_ AR subtypes [31]). Thus, two approaches were adopted to construct the neuronal metabolic profiles, the first to divide the whole-brain samples into striata “only” samples and whole-brain samples that excluded the striata. The second approach investigated the neuronal metabolic profile of significant metabolites in whole-brain (excluding striata) and striata samples via untargeted metabolomics performed through ^1^H-NMR and GC-TOFMS analyses.

#### 3.2.1. Striatal Metabolic Alterations

As described throughout Section 2, most of the statistically significantly altered metabolites were decreased in rats treated with compound **1a** and reference compounds compared to the control group, similar to the serum samples. However, in contrast to the serum samples, very few amino acid alterations were observed, with lipid metabolites comprising most of the metabolites with statistically significant alterations.

Interestingly, KW-6002 exhibited little effect on the striata, with only two metabolites presenting statistically significantly altered metabolites, despite the striata being a primary target of the selective A_2A_ AR antagonist. Based on the serum metabolic profile of KW-6002, a reduced level of the statistically significant metabolite myo-inositol was found. Myo-inositol, an essential precursor for the phosphoinositide pathway, was previously documented to be decreased after fluoxetine (a known antidepressant drug) treatment in the hippocampus [29]. In analogy to the latter study, it is speculated that KW-6002 may exert its therapeutic effect via the phosphoinositide pathway, which is involved in signaling in serotonergic neurotransmission [29].

Nevertheless, compound **1a** and imipramine presented promising results. Firstly, concerning energy metabolism, compound **1a** and imipramine exhibited reduced pyruvate levels compared to the control group. Pyruvate is the main metabolic end-product of glucose metabolism (when metabolized via glycolysis) and the main driver of the Krebs cycle. It is well known that the brain is a very energy-intensive organ, consuming 20–25% of the body’s glucose daily [32]. Of the 14 metabolites that overlapped between compound **1a** and imipramine, it is worth mentioning pyruvate (see Figure 2c). Since compound **1a** and imipramine’s striata presented reduced pyruvate, it might indicate that their striata had a lower energy demand than the control group, and by extension, it could indicate less stimulation.

Secondly, as mentioned above, lipid metabolism was the most affected by compound **1a** and imipramine. One may ascribe this to perturbations in energy metabolism; however, it is important to note that lipids play different roles within the brain compared to the rest of the body. Direct energy production from lipids, specifically fatty acids via β-oxidation, can produce large amounts of ATP [33]. However, this is accompanied by the production of large amounts of reactive oxygen species (ROS), to which the brain is susceptible and easily damaged. Consequently, these alterations being caused by energy metabolism perturbations is highly unlikely. A much more likely explanation for them could be treatment-induced striatal self-repair mechanisms, with the lipids exhibiting reduced levels having been used to repair neuronal cell membranes. Examples could include stearic acid (compound **1a** and imipramine), palmitic acid (compound **1a** and imipramine), oleic acid (compound **1a** and imipramine), linoleic acid (compound **1a** and imipramine) [34], as well as myristic acid (compound **1a**) [35], glycerol (compound **1a**) [30], and cholesterol (KW-6002 and imipramine) [36]. The latter is in accordance with previous studies examining an antidepressant drug’s metabolic effects. The authors described decreased brain lipid levels and ascribed them to self-repair mechanisms [30].

However, certain lipids have functions beyond energy sources or membrane structural components. For instance, poly-unsaturated fatty acids (PUFAs) have various roles as bioactive molecules, especially pertaining to inflammatory responses in the brain, and many of them are prostaglandin precursors [33]. Omega-6 fatty acids, such as linoleic and arachidonic acid, generally exhibit pro-inflammatory activity, while omega-3 fatty acids, such as docosahexaenoic acid, display anti-inflammatory activity. Linoleic acid was decreased in compound **1a** and imipramine’s striata samples, indicating their probable anti-inflammatory activity. However, docosahexaenoic acid also presented reduced levels. Compound **1a** and imipramine may exert an anti-inflammatory effect; however, this warrants further investigation.

In analogy to inflammation, compound **1a** and imipramine may exert neuroprotective activity by reducing oxidative stress. The latter is based on the decreased striatal levels of mainly ribitol and pyroglutamic acid. Ribitol has been shown to increase oxidative stress/ROS production [37] and can be used to indicate cellular oxidative stress levels. Furthermore, pyroglutamic acid is an intermediate in glutathione metabolism, produced when glutathione is broken down (i.e., oxidized by ROS). Therefore, the higher the oxidative stress load, the higher pyroglutamic acid concentrations [38]. Since compound **1a** and imipramine’s ribitol and pyroglutamic acid levels were reduced compared to the control group, it is plausible that they exert a neuroprotective function in the striata via the reduction of oxidative stress.

Finally, another noteworthy metabolite that presented decreased levels was 3,4-dimethoxy mandelic acid (overlap between compound **1a** and imipramine). Mandelic acid and its derivatives are produced during the metabolism of epinephrine and/or norepinephrine [39]. The latter could indicate reduced epinephrine and/or norepinephrine activity/release by extension, since their downstream metabolites were reduced. If this is considered in terms of norepinephrine, compound **1a** and imipramine may act as norepinephrine reuptake inhibitors in the striata. This is a known mechanism of action of tricyclic antidepressants such as imipramine and could indicate a potential antidepressant mechanism of action for compound **1a**. However, this is only speculative at this stage and would require more in-depth research to confirm.

#### 3.2.2. Whole-Brain (Excluding Striata) Metabolic Profiles

In contrast to the decreased metabolite levels observed with the serum and striata samples, the whole-brain (excluding striata) samples exhibited statistically significant metabolites, with increased levels compared to the control group for compound **1a** and reference compounds. Noteworthy, the whole-brain (excluding striata) samples possessed significant alterations of lipid metabolism in comparison to the control group. Given that the brain maintains the second-highest concentration of lipids in the body [33], this is no surprise.

A previous study by Zhao et al. [34] also aimed to metabolically profile imipramine’s effects in the brains (hippocampus) of rodents subjected to the FST (though these were mice of the chronic unpredictable mild stress depression model). They were able to identify 13 perturbed metabolites, of which five were also identified in the current study, namely aspartic acid, *N*-acetyl aspartic acid, stearic acid, palmitic acid, and oleic acid. The three identified fatty acids (stearic, palmitic, and oleic acid) were also higher in the groups treated with imipramine than the control groups, similar to the alterations observed in the current study. However, Zhao *et al.* [34] found that aspartic acid and *N*-acetyl aspartic acid levels were higher and lower, respectively, in the imipramine-treated groups, while the contrary was exhibited in the current study. This contradiction may be ascribed to the different brain regions used to construct the metabolic profiles.

As explained in Section 3.2.1, it is doubtful that the alterations are due to perturbations in energy metabolism. Considering the vital role that lipids play as structural elements in neuronal cell membranes, increasing metabolite levels could improve recruitment originating from ongoing self-repair mechanisms. The increased recruitment may be reflected in the increased levels of palmitic acid and, to a lesser degree, stearic acid (exhibited by **1a** and reference compounds), which are considered the metabolic end-products of fatty acid synthesis. Ongoing self-repair mechanisms would require increased lipids used for these repairs, thus warranting increased synthesis. Consequently, this indicates possible neuroprotective effects induced by **1a** and reference compounds.

Furthermore, lipids’ roles beyond cell membrane structural components need to be considered. Many lipids function as bioactive molecules, with PUFAs having notable roles in inflammatory responses (see Section 3.2.1). When compared to the control group, **1a** and the reference compounds presented increased levels of docosahexaenoic acid and eicosapentaenoic acid, which should assist in both reducing inflammation and creating an anti-inflammatory environment (since omega-3 fatty acids possess anti-inflammatory activity [33]). However, arachidonic acid, an omega-6 fatty acid, exhibited increased levels. Despite omega-6 fatty acids having pro-inflammatory activity, this increase does not necessarily indicate increased inflammation. Arachidonic acid is a multifaceted metabolite, acting as the precursor to many eicosanoids (such as prostaglandins), as well as the endocannabinoids [40]. Its product prostaglandins are potent promotors of acute inflammatory responses [33], while the endocannabinoids play essential roles in the modulation of emotion, reward and motivation signaling, stress responses, etc. [40]. Since arachidonic acid (metabolite of compound **1a**, KW-6002, and imipramine) levels are increased, it may suggest that less was metabolized to these potent pro-inflammatory prostaglandins, thus contributing to an environment with less inflammation (following the increased omega-3 fatty acids).

In conjunction with reduced inflammation, both compound **1a** and imipramine exhibited reduced pyroglutamic acid levels similar to the striata, possibly indicating reduced oxidative stress. As mentioned in Section 3.2.1, pyroglutamic acid is a downstream metabolite of glutathione. Thus, reduced levels of pyroglutamic acid indicate reduced oxidative stress, seeing as less glutathione was broken down. Reduced glutathione consumption could also be supported by the increased levels of cysteine (observed in **1a**, KW-6002, and imipramine) and serine (observed in KW-6002). Cysteine is a key precursor to glutathione synthesis [41], while serine can be converted to glycine, which is also necessary for glutathione synthesis [42]. These increased levels could thus indicate reduced recruitment for glutathione synthesis, further supporting the possibility of reduced glutathione breakdown.

In addition to the abovementioned evidence for **1a** and reference compounds’ speculated neuroprotective activity, the compound **1a** and imipramine also presented with increased *N*-acetyl aspartic acid levels. *N*-acetyl aspartic acid is the most abundant metabolite unique to the central nervous system (CNS) and has been found to be reduced in various neurological problems. For example, following traumatic brain injury, levels sometimes increase after recovery [43]. The fact that two of the investigated treatment groups presented higher *N*-acetyl aspartic acid levels than the control group could thus further support the idea of their neuroprotective capabilities.

Other noteworthy observations besides **1a**’s potential neuroprotective effects include the altered levels of arachidonic acid. Arachidonic acid is the metabolic precursor of the endocannabinoids, which have very short half-lives, seeing as they are rapidly metabolized upon entering a cell and consequently not stored in vesicles [44]. An increased pool of arachidonic acid could boost endocannabinoid levels when needed since more of their precursor is available to be used in their production. Given their role in modulating mood, emotion, motivation, etc., it is possible that by having the ability to produce more thereof, they could assist in alleviating depressive symptoms.

## 4. Materials and Methods

### 4.1. Ethics

**Figure 5:**
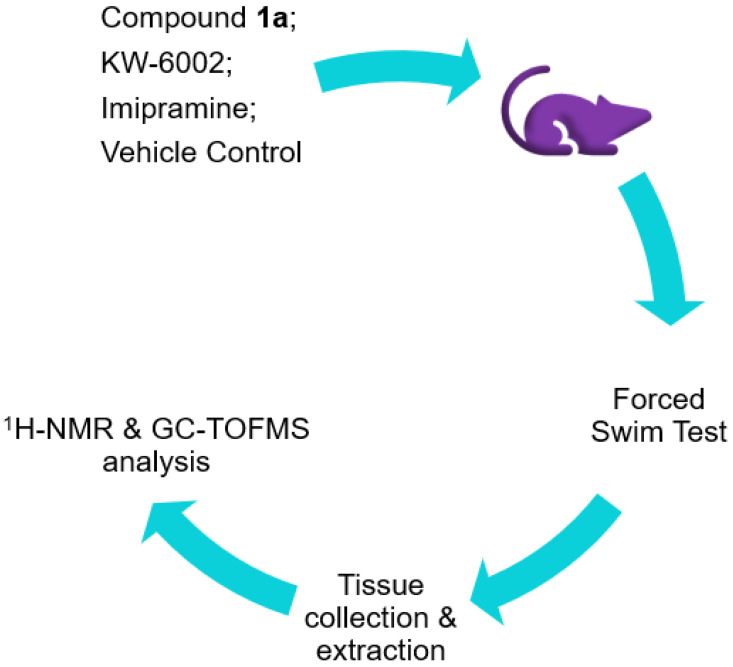
Graphical summary of the applied methodology.

Ethics approval for the study was granted by the North-West University Animal Care, Health and Safety Research Ethics Committee (NWU-AnimCareREC) (Ethics number: NWU-00416-21-A5). A summary of the methodology is provided in Figure 5.

### 4.2. Animal Model and Sampling

Samples used in the current study were acquired from an in vivo study where male Sprague-Dawley rats were given either the vehicle (5% DMSO solution) or treated with compound **1a** (1, 2.5, 5 and 10 mg/kg) or reference compounds (KW-6002, 1.25 or 2.5 mg/kg, and imipramine, 10 mg/kg) and subjected to the forced swim test, as previously described [45]. These treatments were administered via oral gavage for a period of 2 weeks, with the final administration occurring no longer than 18 h prior to euthanization. The test animals were housed in individually ventilated cages (300 mm × 300 mm × 180 mm) with 2–3 rats per cage and *ad libitum* food and water. Environmental conditions were as follows: temperature of 21 ± 1 °C, relative humidity at 55 ± 10%, the light intensity of 400 lux, and a 12 h/12 h light/dark cycle.

Whole-blood and whole-brain material was collected directly following the decapitation of the rats. Whole blood was collected in Vacucare SST gel blood collection tubes kept on ice until serum isolation, which involved centrifuging the whole blood at 2000× *g* for 10 min at 4 °C. The serum was subsequently removed and stored at −80 °C until further use. After collection of the whole-brain samples, the samples were dissected to separate the striata from the whole brain. Hereafter, the striata and whole-brain (excluding striata) samples were separately snap-frozen using liquid nitrogen and stored at −80 °C until further use.

### 4.3. GC-TOFMS Sample Preparation and Analysis

#### 4.3.1. Serum Deproteinization

Deproteinization of the serum samples was carried out as previously described [46]. In short, this involved adding 150 µL ice-cold acetonitrile and 25 µL internal standard solution (3-phenyl butyric acid/nonadecanoic acid solution, final concentration of 25 ppm) to 50 µL serum. After 10 min of incubating on ice, the samples were centrifuged at 12,000× *g* for 5 min at 4 °C. The resultant supernatant was collected, dried overnight under N_2_, and stored at −80 °C until further use.

#### 4.3.2. Metabolome Extraction from Brain Tissue Samples

A small piece (30–70 mg) of the frozen whole brain (excluding striata) was cut from the same area using a clean scalpel and weighed, while the frozen striata samples’ weights were simply noted. The metabolomes of the aforementioned samples were extracted employing a biphasic Bligh-Dyer, as described by Lindeque et al. [46]. Internal standards added to these samples included 3-phenyl butyric acid and nonadecanoic acid to a final concentration of 300 ppm. Following the extraction, 240 µL was taken from the top aqueous phase and 180 µL from the bottom organic phase of each of the striata sample’s extracts. Similarly, 320 µL was taken from the top aqueous phase and 240 µL from the bottom organic phase of each of the whole-brain (excluding striata) sample’s extracts. Each of these aliquots were again split in half (to allow for multi-platform analyses), dried overnight under N_2_, and stored at −80 °C until further use.

#### 4.3.3. Derivatization: Oximation and Silylation

All samples were derivatized prior to GC-TOFMS analysis. This included the dried serum, whole brain (excluding striata), and striata samples. MOX (200 mg methoxyamine dissolved in 10 mL pyridine) was used as an oximation reagent, while BSTFA (containing 1% TMCS) acted as the silylation reagent. Oximation was achieved by adding 50 µL MOX to the dried samples and incubating them for 1 h at 60 °C. Silylation was performed directly after adding 50 µL BSTFA to the samples and again incubating them for 1 h at 40 °C.

#### 4.3.4. GC-TOFMS Analysis

The GC-TOFMS system consisted of an Agilent 7693 autosampler and a 7890 GC system, coupled with a Leco Pegasus HT mass analyzer. The serum, whole-brain (excluding striata), and striata samples were analyzed using the same GC-TOFMS parameters. A Restek RXi^®^-5 column (20 m × 0.2 mm × 0.18 μm) was used for the chromatographic separation, with 1µL sample being injected per run using a split/splitless injector with a split ratio of 1:10. The oven temperature was initially set to 50 °C, which was maintained for 1 min, and subsequently ramped as follows: 5 °C/min to 100 °C, 10 °C/min to 160 °C, 13 °C/min to 230 °C. Total run time thus came to roughly 28 min per sample. Other system temperatures included the inlet temperature at 250 °C, the transfer line at 225 °C, and the source at 200 °C. Lastly, helium was used as the carrier gas with a constant flow of 1.4 mL/min; a solvent delay of 110 s was implemented before data acquisition, which was performed at a rate of 20 spectra (50–950 *m/z*) per second.

#### 4.3.5. GC-MS Data Extraction and Processing

GC-TOFMS data extraction was performed using ChromaTOF (Leco, St. Joseph, MI, USA) according to Venter et al. [47]. This entailed baseline subtraction, peak detection, and deconvolution, with smoothing parameters selected by the software. In order to be identified as true peaks, potential peaks had to have had a peak width of minimum three seconds, a signal-to-noise ratio of at least 20, and at least five apexing masses. Identification was performed using spectral matching to the NIST11 commercial library and an in-house-created library. Identification criteria were set at a spectral match of 80% similarity (akin to 800 in ChromaTOF). Produced peaks were normalized against the internal standard (3-phenyl butyric acid) to obtain the associated metabolites’ concentrations, after which Metaboanalyst was used to perform statistical analysis and create the box-plots seen in Figure 3 [48]. This included a one-way ANOVA analysis of the data. Using Fisher’s HSD results, metabolites with statistically significant differences between the experimental and control groups were identified. The highest dosage groups showed the highest number of metabolites with statistically significant alterations; thus, they were used exclusively (see Supplementary Tables S1 and S2 for a comprehensive list of metabolites). These metabolites were then manually investigated using ChromaTOF to confirm their identities and level of confidence [24]. Furthermore, the Venn diagrams seen in Figure 2 were created using InteractiVenn [49].

### 4.4. ^1^H-NMR Sample Preparation and Analysis

#### 4.4.1. Serum, Whole-Brain (Excluding Striata), and Striata Sample Preparation

To remove macromolecules such as proteins from the serum samples, which could potentially interfere with the ^1^H-NMR analysis, 100 µL of each serum sample was first filtered using pre-rinsed centrifugal filter units (10 kDa pore size). Following this, the serum samples were prepared using a miniaturized ^1^H-NMR method, as described previously [50]. This involved adding NMR buffer solution to the serum samples and transferring them to 2 mm NMR tubes.

In contrast, the preparation of the whole-brain (excluding striata) and striata samples was conducted using the ^1^H-NMR method described by Mason et al. [51]. Briefly, dried aqueous phase extracts were reconstituted using Milli-Q water, to which NMR buffer solution was added and then transferred to 5 mm NMR tubes.

#### 4.4.2. ^1^H-NMR Analysis

The serum, whole-brain (excluding striata), and striata samples were analyzed using the same NMR parameters. Analyses were performed using a Bruker Avance III HD 500 MHz NMR spectrometer equipped with a 5 mm triple-resonance inverse (TXI) probe head. Prior to each analysis, the probe was set to lock onto the deuterated compound (D_2_O) in the NMR buffer, tune itself to the NMR’s magnetic field (500.133 MHz), shim to the Trimethylsilyl-2,2,3,3-tetradeuteropropionic acid (TSP) internal standard (also contained in the NMR buffer), as well as calibrate each pulse to a resonance frequency at 90°. This pre-process helped to ensure that highly reproducible data were produced. Each analysis consisted of 128 scans, each comprising an 8 μs excitation pulse of 90° with a subsequent 4 s relaxation delay.

#### 4.4.3. ^1^H-NMR Data Extraction and Processing

Each brain sample analyzed yielded an NMR spectrum with a width of 12,000 Hz (24.0 ppm), while each serum sample’s spectrum had a width of 6000 Hz (12 ppm). Data pre-processing was performed using Bruker Topspin (V3.5) (Bruker, Billerica, MA, USA), the technicalities of which have been described previously [50]. Further data analysis and processing were carried out using Bruker Amix (V3.9.14) (Bruker, Billerica, MA, USA) and consisted of normalizing all peaks to TSP and dividing the spectrum into bins with a width of 0.02 ppm. The bins containing the spectra’s water regions, which spanned 4.5257–5.8898 ppm for the brain samples and 4.6703–5.1782 ppm for the serum samples, were subsequently removed before statistics were performed. Metaboanalyst was used for statistical analysis of the produced bins, which included one-way ANOVA analysis of the data, as well as the creation of the box-plots seen in Figure 3 [48]. Using Fisher’s HSD results, bins with statistically significant differences between the experimental and control groups were identified. These bins were then manually investigated using Bruker Amix. The peaks present within these bins were subsequently compared to reference compounds in Amix’s commercial library to identify the relevant metabolite, with those of the highest dosage groups used for data interpretation.

## 5. Conclusions

In conclusion, compound **1a** displayed similar metabolic profiles to imipramine in all of the examined matrices (see Figure 2a–c), as well as to KW-6002 in all but the striata (see Figure 2a,b). Notably, the downstream effects on amino acid and lipid metabolism were observed in the treatment groups. Collectively, the test compound **1a** may exert a therapeutic effect based on the shared metabolic profiles with the reference compounds, i.e., systemically by correcting perturbed energy metabolism and neuronally by exerting neuroprotective actions, inhibiting norepinephrine reuptake (in the striata), and/or increasing available arachidonic acid (Section 3). The advantage that compound **1a** holds over traditional antidepressants, such as imipramine, lies in the fact that it belongs to a different drug class, making it a valuable alternative treatment, especially for patients presenting treatment-resistant depression. However, the exact mechanism of compound **1a** remains unclear, and future studies are encouraged to clarify the precise mechanisms of action.

## Figures and Tables

**Table 1:** Compound **1a**’s metabolic alterations in serum and brain samples ^‡^.

Associated Metabolism	Metabolite ^+^	*p*-Value	Alteration		
			Whole Brain ^α^	Striata ^δ^	Serum
Amino Acid	Methionine	0.0018			**↓**
Amino Acid	Isoleucine	0.002			**↓**
Amino Acid	Leucine	0.003			**↓**
Amino Acid	Proline	β *			**↓**
Amino Acid	Glutamine	0.009			**↓**
Amino Acid	Valine	0.007			**↓**
Amino Acid	Cysteine	0.0032	**↑**		
Amino Acid	Norleucine	0.0019	**↑**		
Amino Acid	Glycine	0.0022		**↓**	
Amino Acid	Aspartate	β *; 0.004; β *	**↓**	**↓**	**↓**
Amino Acid	2-OH Butyrate	0.0026			**↓**
Amino Acid	Creatine	0.0004			**↓**
Amino Acid	Creatinine	0.0029			**↓**
Amino Acid	*N*-Acetylaspartic Acid (2)	β *	**↑**		
Amino Acid	Aminoisobutyric Acid (2)	β *	**↑**		
Amino Acid	Glutamic Acid	0.018			**↓**
Lipid	Stearic Acid	0.019; 0.006; β *	**↑**	**↓**	**↓**
Lipid	Palmitic Acid	β *; 0.013; β *	**↑**	**↓**	**↓**
Lipid	Linoleic Acid	0.0014; 0.0081		**↓**	**↓**
Lipid	Cholesterol	0.013			**↓**
Lipid	Gondoic Acid (11-Eicosenoic Acid)	0.0066	**↑**		
Lipid	Eicosapentaenoic Acid (cis-5,8,11,14,17)	β *	**↑**		
Lipid	Arachidonic Acid	β *	**↑**		
Lipid	Docosahexaenoic acid (cis-4,7,10,13,16,19)	β *; 0.012	**↑**	**↓**	
Lipid	Oleic Acid	β *; β *	**↑**	**↓**	
Lipid	Nonadecanoic acid	β *	**↓**		
Lipid	Unknown Unsaturated C18 (2)	β *		**↓**	
Lipid	3,4-Dimethoxy Mandelic Acid	β *		**↓**	
Lipid	Unknown Unsaturated C20 (3)	0.0053		**↓**	
Lipid	Decanoic Acid (2)	0.0046		**↓**	
Lipid	Glycerol	0.0033		**↓**	
Lipid	Myristic Acid	0.0016		**↓**	
Lipid	Phospholipid Segment	β *	**↑**	**↑**	
Lipid	Free Phosphate	0.0012	**↑**		
Lipid	Phosphate (Silanol)	β *; β *	**↑**	**↓**	
Glutathione	Pyroglutamic Acid	β *; 0.0016	**↓**	**↓**	
Inflammation	Prostaglandin g2f alpha (3)	β *		**↓**	
Pentose Phosphate	Ribitol (2)	0.0038		**↓**	
Steroid Hormone	Pregn-4-ene-3,11,20-trione (3)	0.0043		**↑**	
Central Carbon	Lactate	β *			**↓**
Central Carbon	Acetate	β *			**↓**
Central Carbon	Pyruvate	β *		**↓**	
Central Carbon	Citric Acid	0.0036			**↓**
Urea	Urea	0.009			**↓**
Microbiota	TMA	0.0047			**↓**
Microbiota	Benzene (3)	β *	**↑**		
Other	Oxalic Acid (3)	β *	**↑**		
Other	Aminoethylphosphonate	0.0037		**↓**	
Other	Pyrogallol	β *		**↓**	

* = *p* < 0.0009; **^‡^** = metabolites altered in the highest dosage groups (see Section 4); **^+^** = number in brackets indicates metabolite ID levels [24]; **^α^** = whole-brain (excluding striata); **^δ^** = striata.

**Table 2:** KW-6002 metabolic alterations in serum and brain samples ^‡^.

Associated Metabolism	Metabolite ^+^	*p*-Value	Alteration		
			Whole Brain ^α^	Striata ^δ^	Serum
Amino Acid	Methionine	0.0018			**↓**
Amino Acid	Glutamic Acid	0.018			**↓**
Amino Acid	Proline	β *			**↓**
Amino Acid	Valine	0.007			**↓**
Amino Acid	Cysteine	0.0032	**↑**		
Amino Acid	Norleucine	0.0019	**↑**		
Amino Acid	Serine	β *	**↑**		
Amino Acid	Aspartate	β *			**↓**
Amino Acid	Creatinine	0.0029			**↓**
Amino Acid	Aminoisobutyric acid (2)	β *	**↑**		
Lipid	Stearic Acid	β *		**↓**	**↓**
Lipid	Linoleic Acid	0.0081			**↓**
Lipid	Cholesterol	0.006; 0.013		**↓**	**↓**
Lipid	Gondoic Acid (11-Eicosenoic Acid)	0.0066	**↑**		
Lipid	Eicosapentaenoic Acid (cis-5,8,11,14,17) (3)	β *	**↑**		
Lipid	Arachidonic Acid	β *	**↑**		
Lipid	Docosahexaenoic acid (cis-4,7,10,13,16,19)	β *	**↑**		
Lipid	Palmitic Acid	β *	**↑**		
Lipid	Oleic Acid	β *	**↑**		
Lipid	Nonadecanoic acid	β *	**↓**		
Lipid	Phosphate	β *			**↑**
Lipid	Phospholipid Segment	β *	**↑**		
Lipid	Phosphate (Silanol)	β *	**↑**		
Central Carbon	Lactate	β *			**↓**
Urea	Urea	0.009			**↓**
Microbiota	TMA	0.0047			**↓**
Microbiota	Benzene (3)	β *	**↑**		
Other	Myo-Inositol	β *			**↓**
Other	Oxalic Acid (3)	β *	**↑**		
Other	Pyrogallol	β *		**↓**	

* = *p* < 0.0009; **^‡^** = metabolites altered in the highest dosage groups (see Section 4); **^+^** = number in brackets indicates metabolite ID levels [24]; **^α^** = whole brain (excluding striata); **^δ^** = striata.

**Table 3:** Imipramine metabolic alterations in serum and brain samples.

Associated Metabolism	Metabolite ^+^	*p*-Value	Alteration		
			Whole Brain ^α^	Striata ^δ^	Serum
Amino Acid	Cysteine	0.0032	**↑**		
Amino Acid	Norleucine	0.0019	**↑**		
Amino Acid	3-OH Isovaleric acid	0.0015			**↑**
Amino Acid	Aspartate	β *; 0.004	**↓**	**↓**	
Amino Acid	*N*-Acetylaspartic Acid (2)	β *	**↑**		
Amino Acid	Aminoisobutyric Acid (2)	β *	**↑**		
Carbon	Glycolic Acid	β *			**↑**
Lipid	Stearic Acid	0.019; 0.006; β *	**↑**	**↓**	**↓**
Lipid	Palmitic Acid	β *; 0.013; β *	**↑**	**↓**	**↓**
Lipid	Linoleic Acid	0.0014; 0.0081		**↓**	**↓**
Lipid	Cholesterol	0.006; 0.013		**↓**	**↓**
Lipid	Gondoic Acid (11-Eicosenoic Acid)	0.0066	**↑**		
Lipid	Eicosapentaenoic Acid (cis-5,8,11,14,17) (3)	β *	**↑**		
Lipid	Arachidonic Acid	β *	**↑**		
Lipid	Docosahexaenoic acid (cis-4,7,10,13,16,19)	β *; 0.012	**↑**	**↓**	
Lipid	Oleic Acid	β *; β *	**↑**	**↓**	
Lipid	Nonadecanoic acid	β *	**↓**		
Lipid	Unknown Unsaturated C18 (2)	β *		**↓**	
Lipid	3,4 Dimethoxy Mandelic Acid	β *		**↓**	
Lipid	Unknown Unsaturated C20 (3)	0.0053		**↓**	
Lipid	Butyric Acid	0.0056		**↓**	
Lipid	Ethanimidic Acid (3)	0.013		**↓**	
Lipid	Phospholipid Segment	β *	**↑**		
Lipid	Free Phosphate	0.0012	**↑**		
Lipid	Phosphate (Silanol)	β *	**↑**		
Glutathione	Pyroglutamic Acid	β *; 0.0016	**↓**	**↓**	
Inflammation	Prostaglandiene g2f alpha (3)	β *		**↓**	
Pentose Phosphate	Ribitol (2)	0.0038		**↓**	
Central Carbon	Pentose Sugar	0.013			**↑**
Central Carbon	Pyruvate	β *		**↓**	
Central Carbon	Citric Acid	0.0036			**↓**
Microbiota	Benzene (3)	β *	**↑**		
Other	Oxalic Acid	β *	**↑**		
Other	Aminoethylphosphonate	0.0037		**↓**	

* = *p* < 0.0009; **^+^** = number in brackets indicates metabolite ID levels [24]; **^α^** = whole brain (excluding striata); **^δ^** = striata.

## Data Availability

Please refer to suggested Data Availability Statements in section “MDPI Research Data Policies” at https://www.mdpi.com/ethics (accessed on 22 March 2021).

## Supplementary Materials

The following supporting information can be downloaded at: https://www.mdpi.com/article/10.3390/molecules27072094/s1, Table S1: Compound **1a**’s metabolic alterations in serum and brain samples. Table S2: KW-6002 metabolic alterations in serum and brain samples.

## Sample Availability

Samples of the test compound **1a** are available from the authors.
