# Supplementary material for: Untargeted Metabolomics Reveals the Potential Antidepressant Activity of a Novel Adenosine Receptor Antagonist
Source: Molecules. 2022 Mar 24;27(7):2094. doi: 10.3390/molecules27072094 (PMC9000263; doi:10.3390/molecules27072094)
Supplement: Supplementary file 1 [file molecules-27-02094-s001.zip › molecules-1600295-supplementary.pdf]

Supplementary Material

Untargeted Metabolomics Reveals the Potential Antidepressant Activity of a Novel Adenosine Receptor Antagonist

Arnold P. Smith [1], Jeremie Z. Lindeque [1] and Mietha M. van der Walt [1,*]

Table S1. Compound 1a's metabolic alterations in serum and brain samples[‡].

Associated Metabolism	Metabolite (ID confidence level)	p-Value	Dosage groups[+]		
			Whole Brain[α]	Striata[δ]	Serum
Amino Acid	Methionine	0.0018			b; d
Amino Acid	Serine	0.00032	a; b		
Amino Acid	Isoleucine	0.002			b
Amino Acid	Leucine	0.003			D
Amino Acid	Proline	β*			c; d
Amino Acid	Glutamine	0.009			c; d
Amino Acid	Valine	0.007			c; d
Amino Acid	Cysteine	0.0032	b; c; d		
Amino Acid	Norleucine	0.0019	a; b; c; d		
Amino Acid	Glycine	0.0022		a; b; c; d	
Amino Acid	Aspartate	β*; 0.004; β*	b; c; d	d	a; b; c; d
Amino Acid	2-OH Butyrate	0.0026			c; d
Amino Acid	Creatine	0.0004			c; d
Amino Acid	Creatinine	0.0029			c; d
Amino Acid	N-Acetylaspartic Acid (2)	β*	c; d		
Amino Acid	Aminoisobutyric Acid	β*	a; b; c; d		
Amino Acid	Glutamic Acid	0.018			a; d
Lipid	Stearic Acid	0.019 ; 0.006; β*	a; b; d	a; b; d	a; b; c; d
Lipid	Palmitic Acid	β* ;0.013;0.00017	a; b; c; d	b; d	a; b; c; d
Lipid	Linoleic Acid	0.0014;0.0081		d	a; b; c; d
Lipid	Cholesterol	0.013			a; b; c; d
Lipid	Gondoic Acid (11-Eicosenoic Acid)	0.0066	a; b; d		
Lipid	Eicosapentaenoic Acid (cis-5,8,11,14,17)	β*	a; b; c; d		
Lipid	Arachidonic Acid	β*	a; b; c; d		
Lipid	Docosahexaenoic acid (cis-4,7,10,13,16,19)	β* ;0.012	a; b; c; d	a; d	
Lipid	Oleic Acid	β* ;0.0008	a; b; c; d	d	
Lipid	Unknown Unsaturated C18 (2)	0.00082		a; d	
Lipid	3,4-Dimethoxy Mandelic Acid	β*		d	
Lipid	Unknown Unsaturated C20 (3)	0.0053		d	
Lipid	Decanoic Acid (2)	0.0046		d	

Lipid	Glycerol	0.0033		d	
Lipid	Myristic Acid	0.0016		d	
Lipid	Phospholipid Segment	8.09 x10-12	a; b; c; d	a; b; c; d	
Lipid	Free Phosphate	0.0012	a; b; c; d		
Lipid	Phosphate (Silanol)	β*;0.00069	a; b; c; d	d	
Glutathione	Pyroglutamic Acid	β*;0.0016	b; c; d	d	
Inflammation	Prostaglandin g2f alpha (3)	0.00039		d	
Pentose Phosphate	Ribitol (2)	0.0038		d	
Steroid Hormone	Pregn-4-ene-3,11,20-trione (3)	0.0043		b; c; d	
Central Carbon	Pentose Sugar	0.013			a; b; c
Central Carbon	Lactate	β*			c; d
Central Carbon	Acetate	β*			c; d
Central Carbon	Pyruvate	β*		b; c; d	
Central Carbon	Citric Acid	0.0036			c; d
Urea	Urea	0.009			a; b; d
Micriobiota	TMA	0.0047			a; b; c; d
Micriobiota	Benzene (3)	0.00046	a; b; c; d		
Other	Oxalic Acid (3)	1.23 x10-6	a; b; c; d		
Other	Aminoethylphosphonate	0.0037		d	
Other	Myo-Inositol	0.00037			a
Other	Pyrogallol	0.00011		a; b; c; d	

β* = *p* < 0.0009; ‡ = metabolites altered in the highest dosage groups (see Section 4); + = letter indicates dosage groups, a: 1mg/kg, b: 2.5 mg/kg, c: 5 mg/kg, d: 10 mg/kg; *α* = whole brain (excluding striata); δ = striata.

Table S2. KW-6002 metabolic alterations in serum and brain samples.

Associated Metabolism	Metabolite	p-Value	Dosage groups+		
			Whole Brainα	Striataδ	Serum
Amino Acid	Methionine	0.0018			b
Amino Acid	Glutamic Acid	0.018			b
Amino Acid	Proline	β*			a
Amino Acid	Valine	0.007			a; b
Amino Acid	Cysteine	0.0032	b		
Amino Acid	Norleucine	0.0019	a; b		
Amino Acid	Serine	0.00032	b		
Amino Acid	Aspartate	β*			b
Amino Acid	Creatinine	0.0029			b
Amino Acid	N-Acetyl Lysine (3)	0.013			a
Amino Acid	Aminoisobutyric acid (2/3?)	1.82 x10-9	b		
Amino Acid	2-OH Butyrate	0.0026			a
Lipid	Stearic Acid	β*			a; b
Lipid	Linoleic Acid	0.0081			b
Lipid	Cholesterol	0.006; 0.013		b	a; b
Lipid	Gondoic Acid (11-Eicosenoic Acid)	0.0066	a; b		
Lipid	Ethanimidic Acid (Acetamide?) level 3	0.013		a	

Lipid	Eicosapentaenoic Acid (cis-5,8,11,14,17) (level 3?)	β*	a; b		
Lipid	Arachidonic Acid	β*	a; b		
Lipid	Docosahexaenoic acid (cis-4,7,10,13,16,19)	β*	a; b		
Lipid	Palmitic Acid (MG (16:0/0:0/0:0))	β*	a; b		
Lipid	Oleic Acid	β*	a; b		
Lipid	C19? (Tetradecanoic acid)	β*	a; b		
Lipid	Phosphate	β*			b
Lipid	Phospholipid Segment	β*	a; b		
Lipid	Phosphate (Silanol)	β*	a; b		
Central Carbon	Lactate	β*			a; b
Central Carbon	Citric Acid	β*			a
Urea	Urea	0.009			a; b
Other	Myo-Inositol	0.00037			a; b
Microbiota	TMA	0.0047			a; b
Microbiota	Benzene (level 3)	0.00046			
	Aminoethylphosphonate	0.0037		a	
	Oxalic Acid (level 3)	β*	a; b		
	Pyrogallol (gallic acid?)	0.00011		b	

β* = $p < 0.0009$; ‡ = metabolites altered in the highest dosage groups (see Section 4); + = letter indicates dosage groups, a: 1.25 mg/kg, b: 2.5 mg/kg; α = whole brain (excluding striata); δ = striata.
